# Parallel Force Assay for Protein-Protein Interactions

**DOI:** 10.1371/journal.pone.0115049

**Published:** 2014-12-29

**Authors:** Daniela Aschenbrenner, Diana A. Pippig, Kamila Klamecka, Katja Limmer, Heinrich Leonhardt, Hermann E. Gaub

**Affiliations:** 1 Lehrstuhl für Angewandte Physik and Center for Nanoscience (CeNS), Ludwig-Maximilians-; Universität, Amalienstrasse 54, 80799 Munich, Germany; 2 Department of Biology II and Center for Nanoscience (CeNS), Ludwig-Maximilians-Universität, Großhadernerstr. 2, 82152 Planegg-Martinsried, Germany; 3 Munich Center for Integrated Protein Science (CIPSM), Butenandtstr. 5–13, 81377 Munich, Germany; Weizmann Institute of Science, Israel

## Abstract

Quantitative proteome research is greatly promoted by high-resolution parallel format assays. A characterization of protein complexes based on binding forces offers an unparalleled dynamic range and allows for the effective discrimination of non-specific interactions. Here we present a DNA-based Molecular Force Assay to quantify protein-protein interactions, namely the bond between different variants of GFP and GFP-binding nanobodies. We present different strategies to adjust the maximum sensitivity window of the assay by influencing the binding strength of the DNA reference duplexes. The binding of the nanobody Enhancer to the different GFP constructs is compared at high sensitivity of the assay. Whereas the binding strength to wild type and enhanced GFP are equal within experimental error, stronger binding to superfolder GFP is observed. This difference in binding strength is attributed to alterations in the amino acids that form contacts according to the crystal structure of the initial wild type GFP-Enhancer complex. Moreover, we outline the potential for large-scale parallelization of the assay.

## Introduction

Protein-protein interactions are essential to most reactions in the cell and thus their characterization crucial for a better understanding of many fundamental processes in nature [1]. A key problem herein lies in the extensive number of interactions in any given proteome [2]. Several high-throughput methods have been developed to meet this challenge, such as yeast-two-hybrid assays [3], protein microarrays [4], or microfluidic-based techniques [5]. These are valuable tools for the identification of interacting proteins [1], [6]. In addition, several low-throughput methods exist that are able to characterize such interactions in greater detail. Prominent examples, providing different information on the structure or the kinetics of an interaction, are X-ray crystallography [7], fluorescence resonance energy transfer (FRET) [8], or surface plasmon resonance [9]. Another parameter becoming more and more acknowledged is the intermolecular binding force that controls the interaction. Mechanical stability of a biomolecular interaction does not necessarily compare to its thermal stability and *vice versa*. However, mechanical load can for example decrease thermal stability and “off-time” of a bond, which plays a pivotal role in receptor-ligand interactions and thus signaling processes in *e.g.* cell differentiation and immunological recognition. At the other extreme, bonds may be stabilized by exerted forces. These so called “catch bonds” are found across various species and in different biological contexts. In those cases interactions that would otherwise be of transient and low affinity nature are stabilized by the shear force the binding partners experience. Prominent examples are adhesion proteins like integrins [10] and cadherins [11] in humans or FimH [12] in bacteria, which tune their binding properties in response to mechanical stress [13]. Another example for potential biological importance of binding forces is in autoproteolyzed domains of Adhesion-GPCRs, where the two parts of the protein are hypothesized to unbind at a certain force threshold. This could serve as a protective mechanism upon exposure to mechanical stress [14]. As the impact of forces in those contexts is challenging to study it can be assumed that other examples will follow.

In order to address questions regarding forces in biomolecules or biomolecular interactions, single-molecule force spectroscopy techniques have been developed, based on *e.g.* the atomic force microscope (AFM) [15], [16] or optical tweezers [17] enabling direct quantification of the forces and energy landscapes underlying protein-protein interactions [18]–[20]. Common drawbacks of those single-molecule techniques are the high effort needed to gather statistically sufficient data sets or the infeasibility to measure different interactions in parallel, giving rise to calibration uncertainties [21]. Thus, a method able to parallelize force measurements of protein-protein interactions is highly desirable.

As low throughput is a general limitation of force-based single-molecule experiments, our lab has recently developed the Molecular Force Assay (MFA) to overcome this bottleneck. Relying on the principle of comparing the bond in question with a known reference bond, single-molecule measurements can be conducted in parallel. In detail, the two complexes to be compared are attached in series to form a so-called Molecular Force Probe (MFP) upon which a force is applied. The force directly correlates the mechanical stability of both bonds until, statistically, the weaker bond ruptures. In one single experiment thousands of MFPs can be tested simultaneously. Additionally, the sample and reference bond can be multiplexed. This very sensitive method has already been applied successfully to DNA, *e.g.* to resolve single base-pair mismatches [22]. It was further utilized to characterize the binding of ligands like polyamides [23] or proteins [24] to DNA as well as to RNA [25]. In order to enhance the throughput, the capacity of the MFA technique for parallelization, by means of a microfluidic chip [26], as well as for miniaturization [27] has been demonstrated. In a first approach to determine protein interactions, a force-based sandwich immunoassay relying on the basic principle of two bonds in series was constructed [28]. Here, we introduce parallelized force measurements of protein-protein interactions utilizing site-specific and covalent integration of a protein pair into the MFA. Our proof-of-principle study aims to test the binding of three variants of Green Fluorescent Proteins (GFPs) [29] to the GFP-binding nanobody “Enhancer” [30]. To be able to detect the differences in binding strength, first the window of high sensitivity of the assay is determined by testing against references with different binding strengths. In order to highlight the dependence of the sensitivity on the chosen reference, a modified variant of Enhancer, displaying a different binding strength to GFP, is employed and compared to Enhancer.

Nanobodies are camelid-derived single-domain antibodies. Enhancer has been generated and selected for its modulation of the conformation and the spectral properties of wild type GFP (wtGFP), where its binding leads to a fourfold fluorescence enhancement [30]. The binding epitopes of the nanobodies lie on the outer beta barrel structure, which is conserved for the other GFP variants investigated here, namely superfolder GFP (sfGFP) [31] and enhanced GFP (eGFP) [32]. As GFP binding nanobodies are stable and functional in living cells, they have been used for numerous applications. Examples are the detection of translocation events *in vivo* [30], the high affinity capture of GFP fusion proteins [33], or enabling GFP to act as scaffold for the manipulation of gene expression [34]. All rely on the nanobodies' excellent binding specificities. In addition to being well characterized, this system offers the advantage of GFP acting as an intrinsic fluorescence label to control for the correct assembly of the Protein-MFA.

## Results and Discussion

### General Functionality of the Protein Molecular Force Assay

**Figure 1:**
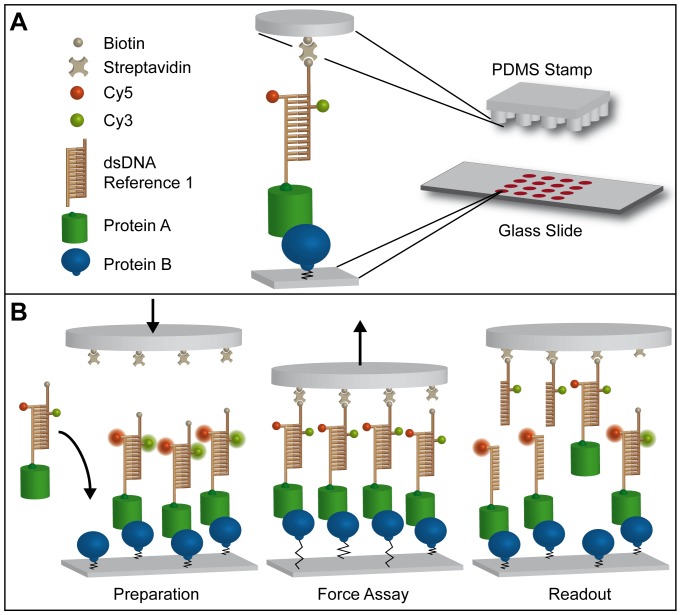
Basic Principle of the Protein Molecular Force Assay. (A) Molecular Force Probes (MFPs) consist of two bonds in series, a protein complex to be studied and a DNA duplex acting as a reference. Both proteins are attached covalently at their N- or C-terminus, one to the glass slide and the other one to a strand of the DNA duplex. Cy5 and Cy3, coupled to one of the DNA strands each, form a FRET pair. Linkage to the upper surface, a PDMS stamp functionalized with Streptavidin, is facilitated *via* a Biotin on the DNA. In the macroscopic view, the PDMS stamp with 16 pillars as well as the glass slide with a matching 4×4 array of spots of MFPs is displayed. Every spot may be functionalized with a different set of MFPs, allowing for the measurement of 16 different protein pairs and/or the variation of the reference. (B) *Preparation:* After the stepwise assembly of the MFPs on the glass slide, fluorescence “Start” images of the Cy5 signal (with red excitation) as well as the FRET of the MFPs are recorded. Assembly of the assay is completed by lowering the stamp, which enables the Biotins of the MFPs to bind to the Streptavidins on the elastomer. *Force Assay*: Upon retraction of the stamp with constant speed, a force is gradually built up in the MFPs, acting equally on all molecular components in series. As a result, either the DNA reference duplex or the protein-protein interaction unbinds, resulting in the transfer of either Cy3 alone or Cy3 together with Cy5 to the surface of the stamp. *Readout*: Another set of fluorescence “Final” images of the glass surface provides the ratio of broken protein to reference bonds. The ratio of the Cy5 signals on the glass slide provides the surface density of remaining, intact protein complexes in comparison to the initial number of protein pairs. The residual FRET signal accounts for complexes that were not loaded under force and are still fully assembled. The ratio of the FRET signal thus allows for the correction of the analysis.

Based on the principle of the standard DNA-MFA [24], the Molecular Force Probes of the Protein-MFA consist of two molecular bonds in series, which are attached between two surfaces. The bond to be probed is the protein complex, where both proteins are attached covalently, one to the glass slide, which acts as the lower surface and the other to one strand of a DNA duplex which acts as the reference bond. A Cy5 dye is attached to the DNA strand coupled to the protein. The complementary DNA strand is labeled with a Cy3 dye, forming a FRET pair with the Cy5, as well as with a Biotin, which enables the coupling to the upper surface, a soft PDMS stamp functionalized with Streptavidin (Fig. 1A). The PDMS stamp has a size of 1 cm ×1 cm and features 16 pillars of 1mm in height and 1.1mm in diameter. A matching 4×4 array of MFPs is assembled on a glass slide, where each spot can be functionalized independently, enabling the measurement of 16 different protein pairs and/or the variation of the reference DNA (Fig. 1A). For the preparation of the measurement, first the lower proteins are attached to the glass slide *via* a PEG linker, then the pre-incubated complex of upper protein and DNA reference is added. Multiple washing steps after each incubation step minimize unspecific binding. Fluorescence “Start” images of the Cy5 (red excitation) and FRET signals are recorded for each spot on the glass slide with an inverted epi-fluorescence microscope. After the stamp is lowered gradually onto the glass slide using reflection interference contrast microscopy [35], an incubation step of 10 min allows for the coupling of the Biotins to the Streptavidin on the stamp. A piezo actuator enables retraction of the stamp with constant speed, gradually building up a force acting on both complexes of the MFPs until, statistically, the weaker one unbinds. Here, the retraction speed of 1 µm/s yields a force loading rate in the range of 10^5^ pN/s [27], [36]. After the retraction of the stamp, another set of “Final” fluorescence images is taken as the ratio of remaining dyes determines the outcome of the experiment.

The Normalized Fluorescence (NF) gives the number of broken upper DNA bonds normalized to the total number of Molecular Force Probes that have been under load. To determine the NF, the “RED“ and “FRET” signals recorded of every single spot before and after the actual force assay are processed after background correction. In the analysis, the ratio of RED Final to RED Start gives the density of still intact protein bonds in comparison to the initial amount of protein bonds.



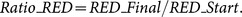



The ratio of FRET values needs to be determined as well, as a remaining FRET signal after the force assay gives the number of MFPs that have not been under load and are thus still fully assembled (see Fig. 1B). For those MFPs, the Cy5 dye giving the RED signal is still attached to the surface yielding a false positive signal. By determining the FRET ratio *(Ratio_FRET  =  FRET_Final/FRET_Start)*, those MFPs can be subtracted.

Normalization to the Coupling Efficiency *CE  = 1– Ratio_FRET* yields the Normalized Fluorescence:


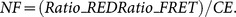
 (Equation 1)

Thus, a NF of 0.5 in this context means that the protein and the DNA complex have the same binding strength, a NF closer to 1 indicates that the protein complex is stronger than its DNA reference and *vice versa* for a NF closer to 0. For the analysis, the assumption is made that all MFPs are correctly assembled in the beginning, meaning that every protein-DNA complex has the second DNA strand attached to it. This is achieved by pre-incubating the DNA in a stoichiometry of 1∶2 before coupling to the protein. If only the lower protein is present with nothing bound to it, it does not give a fluorescent signal and can thus be neglected. The RED and FRET signals cannot be compared directly by division, as the fluorescence efficiency of a Cy5 dye is different to that of a Cy3-Cy5 FRET pair. As demonstrated before by Severin *et al.* [24], the pixel-by-pixel method offers the advantage of canceling out inhomogeneities due to the Gaussian illumination profile or coupling density as well as surface defects. Importantly, in the actual force assay all MFPs are tested simultaneously in the moment of the retraction of the stamp while the read-out can take place subsequently without time constraints [27]. Another very substantial advantage is that the force assay is not disturbed by complex ambients [37] since only fluorescence from the lower surface is measured.

Supporting information on chemical protocols and the measurement process can be found in Materials and Methods in S1 Supplement and S1 Fig.

One of the key challenges in the integration of functional proteins in the MFA was their covalent attachment, especially to the DNA. In principle, different possibilities exist for the coupling of proteins, although differing widely in yield, experimental effort and cost as well as the applicability for attachment to DNA [38], [39]. For the experiments conducted here, as for single-molecule force spectroscopy measurements in general, the site-specific attachment is of utmost importance, as the force needed to unbind a complex depends on the pulling geometry and thus on the position of the attachment [40]. Additionally, to prevent possible mis-assembly, it is reasonable to choose two different strategies for the attachment of the two proteins. In the study presented here, we employed the ybbR-tag [41] on the GFPs' N-termini to covalently attach 5′ Coenzyme A-modified DNA. The coupling is mediated by the Phosphopantetheinyl Transferase Sfp [42], [43] and offers the advantages of very high yield (up to 90%) [44] and a negligible size (11 amino acids) of the protein modification. Further, it has been successfully employed *e.g.* in single-molecule force spectroscopy experiments for the coupling of different proteins in varying experiments to DNA [44], [45] and surfaces [21], [46].

The nanobodies are attached to the glass slide by coupling of the free C-terminal Cysteine to the maleimide group of a heterobifunctional PEG linker [47]. As no extra components are needed, this is a good choice, provided that the protein does not harbor any other accessible or interfering Cysteine residues.

While not needed for the readout of the actual experiment, the use of GFP in this proof-of-principle system offers the advantage of an additional intrinsic control. We observed colocalization of GFP-fluorescence with the fluorescence of the Cy3 and Cy5 dyes, which confirms the specific interaction and correct assembly of the Protein MFPs. The surface density of the Protein MFPs estimated from the Cy5 signal is, similar to previous MFA experiments, about 10^4^ MFPs per µm^2^ [23], [27]. The results for the NF values are reproducible over numerous experiments conducted independently (see S1 Table). However, the most valid conclusions on very small differences can be drawn from data received by a single experiment since it offers exactly the same environment and treatment such as pressure of the stamp and loading rate.

### Adjusting the Sensitivity of the Protein-MFA with Different References

As with an old-fashioned scale, the MFA has its highest sensitivity to discriminate very small differences if it is well balanced, which in our case means that the binding strengths of both complexes are very similar, so that the NF lies close to 0.5. For pure DNA-MFA experiments the strength of the reference could easily be tuned by varying length, composition and conformation of the DNA duplex, reaching from 15pN for DNA in zipper mode (by opening the DNA like a zipper from the same end) [48] to about 65pN by implementing a 40 base-pair (bp) duplex in shear mode (where the DNA is sheared by applying the force at opposing 5′ termini). Higher average forces cannot be reached with short oligonucleotides as DNA reaches a force plateau at about 65pN when sheared due to the so-called BS-transition [49], [50]. Forces in between can be achieved by varying the number of base pairs in shear mode [51]. For a random protein-protein complex, no information is given about the interaction strength *a priori*.

In the study presented here, the tested protein complexes between nanobodies and GFPs were stronger than a 40bp duplex in shear confirmation, resulting in very high NF-values (see S1 Table). To determine small differences in binding strength, higher sensitivity at NF-values closer to 0.5 is highly desirable, which can be obtained by increasing the strength of the reference. To demonstrate the flexibility and robustness of the Protein-MFA, two different methods to enhance the mechanical stability of the DNA reference are presented here.

The stability of the DNA duplex can be altered intrinsically by nucleobase modification, methylation of the 5′ position in cytosines [52], [53] being a prominent example. Studied primarily in duplex formation with RNA for antisense gene inhibition [54], the modification of the 5′ position of pyrimidines with a propynyl group [55] results in an even higher increase in melting temperature than achieved by 5′ methylation [55]-[57]. The propynyl group is planar with respect to the heterocycle and extends into the major groove. It is thus expected to stabilize the duplex due to increased base-stacking and a smaller unfavorable entropy change [55], [57], [58]. In the experiments presented here, a 40bp DNA duplex is employed as a reference, where in the biotinylated strand 13 cytidines and 9 thymines are replaced by their corresponding propynyl bases. In comparison to this intrinsic stabilization, the stability can also be altered extrinsically by the addition of a DNA binding ligand. As has been shown in previous studies with the MFA [23], [59], sequence-specific binding of pyrrole-imidazole hairpin polyamides [60], [61] to the minor groove of the DNA helix enhances the stability of the duplex depending on the modification and concentration of the polyamide. For the experiment presented here, three hairpin polyamides with different affinities for the same DNA sequence have been employed. Polyamides P1 (K_D_  = 105pM), (*R*)-P2 (here P2; K_D_  = 44pM) and (R)-P3 (here P3; K_D_  = 1442pM) described in Ho *et al.* [23] have been used in a concentration of 1 µM, approximately 1000 times higher than the saturation concentration, to ensure an excess of available ligand (see S2 Fig. for the DNA sequences as well as the chemical structures of the propynyl bases and the polyamides). P2 displays higher affinity than the sequence-specific binding P1, as it was modified with an amine substituent to introduce chiral selectivity. P3's lower affinity, despite also being chiral, results from a single base-pair mismatch [23].

**Figure 2:**
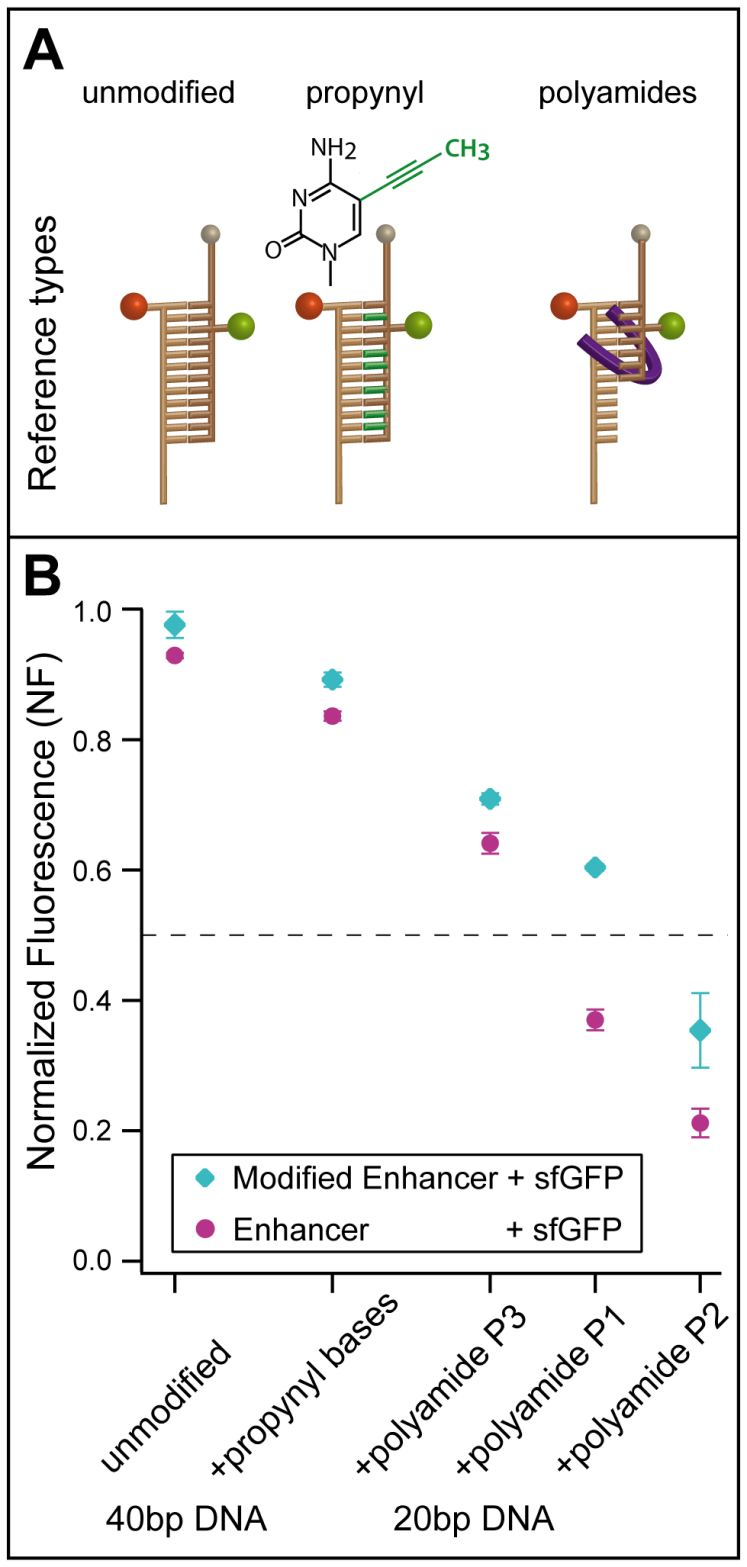
Utilization of Modified Reference DNA Duplexes to Adjust the Sensitivity Window in a Multiplexed Protein-MFA. (A) Three different reference types are compared: unmodified DNA (left), intrinsically stabilized DNA (center), where a part of the pyrimidine bases is replaced by corresponding propynyl bases, as well as extrinsically stabilized DNA (right), where the addition of a specific polyamide ligand [23] enhances the binding strength. (B) Representative sample measurements of Enhancer and Modified Enhancer binding to sfGFP for all types of references are displayed. The NF shows a clear dependence on the reference strength. The NF is higher for the Modified Enhancer than Enhancer in all cases. Additionally, the difference in NF between Modified Enhancer and Enhancer increases the closer the NFs are to 0.5, displaying the higher sensitivity in this range.

Fig. 2A depicts the three different reference types used to identify the window of high sensitivity of the assay: unmodified 40 bp double-stranded DNA, intrinsically stabilized DNA using propynyl bases, and extrinsically stabilized DNA through the binding of sequence-specific polyamide ligands. Representative data for the different references testing Enhancer and Modified Enhancer against sfGFP are depicted in Fig. 2B with standard deviation. The original values can be found in S2 Table. Small differences in the size of the error bars can be attributed to measurement error. For all three types of reference, the outcome of the experiment – namely the relative higher NF values for the Modified Enhancer in comparison to Enhancer – stays the same, but the absolute NF values change depending on the reference. This was to be expected since the reference does not influence the nanobody-GFP complex itself so that the relative ranking of the stability of the complexes stays preserved. Whereas the incorporation of propynyl bases into a 40 bp DNA duplex reduces the NF values about 10%, employing a 20 bp DNA reference with added polyamide ligand leads to larger drops in NF depending on the polyamide. Notably, the closer the mean of the NF values for one reference is to 0.5, the larger the difference between the data points for Enhancer and Modified Enhancer becomes. This is consistent with the higher sensitivity of a well-balanced MFP.

The stronger binding of the Modified Enhancer can be attributed to its more positive charge (pI ≈9.89) when compared to the original Enhancer (pI ≈7.85), as sfGFP is slightly negatively charged (pI ≈6.4) under the given buffer conditions (pH 7.4). This ranking holds also true for the other GFP variants wtGFP (pI  = 6.17) and eGFP (pI  = 6.04), as can be seen in S1 Table. The incorporation of propynyl bases into the 40 bp DNA duplex obviously tunes the molecular balance closer to neutral, but with NF values of approximately 0.8 the result is still not entirely satisfying. Not much is known at present about the molecular mechanisms of the stabilization of the DNA duplex by the propynyl bases. The apolar methyl group is assumed to be buried in the core of the DNA double strand and by means of this contributes to the hybridization energy *via* hydrophobic interaction. Since the increase in stability of the reference depends on number and position of included propynyl bases [62], they represent a versatile tool for fine-tuning the reference bond. Whether this modification of the local interactions results in a change of the potential width or only deepens the potential well is not known yet and will be in the focus of future AFM-based single-molecule force spectroscopy studies.

In comparison to the intrinsic stabilization by propynyl bases, the addition of a polyamide has a much stronger impact on the NF, depending on the chosen polyamide. As expected, the lower the K_D_, the higher the stabilization of the DNA reference and thus the lower the NF. While P3 already has a bigger effect on the NF than the incorporation of propynyl-bases, P1 tunes the MFA closest to neutral. In fact the addition of the polyamide P2 tunes the balance towards the other side resulting in an NF between 0.2 and 0.4. This enables to probe even stronger protein bonds than that of nanobody-GFP complexes. The polyamides used for the given study are known to bind into the minor groove of the DNA, thereby enhancing its mechanical stability, as has been found also for other DNA binding molecules [63], [64]. As shown in Ho *et al*. [23], such polyamides can be designed to modulate the stability of a DNA helix in a wide range. Following this principle, other DNA binding molecules might be candidates to change the DNA reference stability extrinsically as well.

Summarizing, DNA offers the possibility to introduce a very broad range of references with different mechanical and thermal stability, ranging from low forces of about 15 pN with DNA in zipper mode over shear mode DNA in various lengths to enhanced stability *via* intrinsic or extrinsic modification of the DNA. The dynamic range of the mechanical stability of DNA-based references can be extended even further towards higher stabilities by the use of DNA binding proteins such as EcoRI and p53 [27]. Protein-MFA is thus applicable for many different protein pairs of varying bond strength.

### Investigation of the Enhancer-GFP System with Protein-MFA

**Figure 3:**
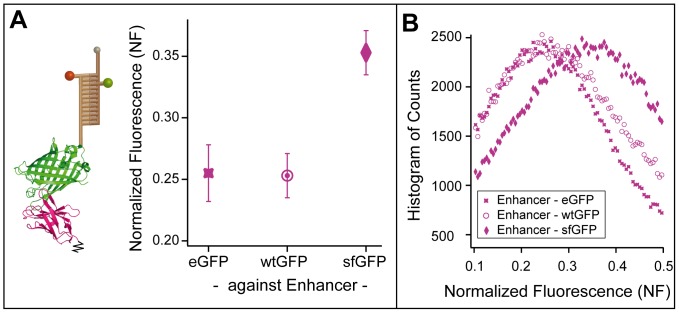
Analysis of Different GFP Variants for Enhancer Interaction Strength with Protein-MFA. (A) Schematic depiction of the MFP for the measurement of the interaction between GFP and Enhancer with the ribbon model structure of wtGFP (green) with Enhancer (magenta) (crystal structure from [30], PDB file 3K1K). One example measurement depicts the differences in binding strength of Enhancer tested against enhanced, wild type, and superfolder GFP with the same reference DNA (20 bp DNA stabilized with polyamide P1). While the binding to eGFP and wtGFP lie within the same range, binding of Enhancer to sfGFP is distinctively stronger. All data points are determined in one single measurement process, derived as the mean of several protein spots and displayed with standard deviation error bars. (B) Sample histograms of MFP spots of Enhancer measured against all three GFP variants illustrate the extensive number of parallelized single-molecule experiments. The Normalized Fluorescence (NF) is determined by dividing the raw fluorescence images before and after transfer pixel-by-pixel (according to Equation 1), and fitting of a Gaussian to the resulting histogram of all pixel counts.

Fig. 3A depicts the result of one representative example measurement, where the binding between the nanobody Enhancer and the three different variants of GFP, namely enhanced GFP, wild type GFP, and superfolder GFP are compared. As shown in the ribbon model structure for wild type GFP [30], all GFP constructs are attached at their N-termini to the DNA reference while Enhancer is coupled to the glass slide *via* its C-terminus. For this measurement, the 20 bp DNA stabilized with polyamide P1 was used as a reference. P1 was chosen as its use could tune the NF in the measurement shown in Fig. 2B closest to neutral. All data points are derived from one contact process with a single stamp ensuring exactly the same conditions and thus minimizing measurement error. As the reference DNA is the same for all protein pairs, comparing the resulting NF values provides information about the differences in the binding strengths of the protein-protein interactions. Displaying the bulk readout of the extensive number of parallelized single-molecule measurements, sample histograms of protein spots with MFPs of all three GFPs are shown in Fig. 3B. In order to evaluate the outcome of the MFA experiment, the Normalized Fluorescence NF is calculated by dividing the fluorescence images according to equation 1. The most-likely NF is then determined by Gaussian fitting of the resulting count histogram.

While the NF values for the Enhancer-eGFP (0.255±0.023) and Enhancer-wtGFP (0.253±0.018) interaction are the same within experimental error, they both lie distinctively lower than the value for the Enhancer-sfGFP (0.353±0.018) construct. This corresponds to a higher ratio of resulting intact Enhancer-sfGFP complexes than Enhancer-eGFP or wtGFP complexes after force application, implying that for this specific pulling geometry the Enhancer-sfGFP interaction is stronger.

From the crystal structure of wtGFP binding Enhancer (PDB 3K1K), Kirchhofer *et al.* [30] determined 9 amino acids that form 13 direct contacts and 3 amino acids forming hydrophobic interactions. The alignment of the amino acid sequences of all three GFP variants (see S3 Fig.) shows that all interacting amino acids of wtGFP are conserved for eGFP, which is in good agreement with the similar binding strength observed in Fig. 3A. The difference in binding strength of sfGFP to Enhancer could result from the mutation of two of the amino acids which form direct contacts to Enhancer and all three amino acids responsible for the hydrophobic interaction. Notably, in force spectroscopy experiments the pulling geometry may have a significant impact on the unbinding force [40].

### Conclusion

With the proof-of-principle system of nanobodies binding to GFPs, we successfully demonstrated the implementation of the Molecular Force Assay in parallelized measurements of protein-protein interactions. The reference strength of the DNA duplex can be adjusted as required both intrinsically through modification of the bases or extrinsically by binding of a ligand to ensure high sensitivity of the assay for the investigated interaction. In addition, the assay has a multiplexing capacity for different protein pairs and provides the high sensitivity and versatility of a fluorescence readout. With a moderate experimental effort, high statistics can be achieved in a single experiment with easy and very fast analysis. The parallel format of the assay also offers the advantage of testing the proteins only once, allowing the measurement of proteins that lose their original conformation upon application of force. With the current set-up, protein interactions that dissociate in the time span of the experiment can not be investigated. A solution would be an alternative set-up of the MFA such as presented in [65], where the upper part of the MFP is attached to the stamp. Also, at the moment only a limited number of protein-pairs can be tested simultaneously and to obtain absolute values the binding strength of the reference has to be known. Additional miniaturization and parallelization will further emphasize the main advantage of the Protein-MFA, namely the high sensitivity due to the comparative approach of the assay. It has already been shown [27], that the results for DNA-MFA do not change when the diameter of the MFP spot is reduced from 1 mm in our current standard set-up to approximately 20 µm. In Otten *et al.* [26] the MFA system was integrated into a microfluidic chip, enabling the measurement of 640 spots of MFPs simultaneously. The next goal will be to combine the parallelization and miniaturization with the expression and direct covalent attachment of the lower protein in a microfluidic chip, as demonstrated recently [21], to turn the Protein-MFA into a high-throughput method. Such a set-up would allow the additional measurement of standardized protein pairs with known rupture force in the same stamping process, which could provide a very robust way to gain even more accurate information about the absolute values of the rupture forces. Creating a “toolbox” of references will render the Protein-MFA applicable to measure an extensive number of protein pairs and a fast way to determine and compare binding strengths.

## Supporting Information

S1 Fig **Coupling of CoA-DNA to ybbR-tagged GFP.** SDS-PAGE gel displaying the coupling between CoenzymeA-modified DNA to the ybbR-sfGFP construct in both fluorescence scans and Coomassie staining. In this sample gel, both GFP and CoA-DNA were mixed in equal concentrations (5 µM) as in the standard protocol [42].
(PDF)

S2 Fig **DNA References.** The reference DNA duplexes are displayed. The strand containing the CoenzymeA and Cy5 modification stays the same for all three types of reference, whereas the complementary strand modified with Cy3 and Biotin varies in length and constitution of bases. Chemical structures of the propynyl bases replacing their corresponding cytidine and thymidine bases are shown (structures provided by biomers.net GmbH, Germany). The polyamide ligands *P1*, *(R)-P2* and *(R)-P3* from [23] bind to the highlighted six base pair long binding sequence in the DNA reference duplex.
(PDF)

S3 Fig **Sequence Alignment of the GFP Variants.** The sequence alignment of all three variants of GFP displays the differences in the amino acid sequences and highlights the positions of the direct contacts (pink) and hydrophobic interactions (pale pink) to the nanobody Enhancer obtained for wtGFP by [30]. For eGFP, none of the interacting amino acids are mutated, but for sfGFP two of the contacts sites for Enhancer are different. In addition, all three amino acids forming the hydrophobic interaction are mutated. Sequence Alignment of GFPs was performed using Clustal W2 (http://www.ebi.ac.uk/Tools/msa/clustalw2/).
(PDF)

S1 Supplement **Materials and Methods.**
(PDF)

S1 Table **Reproducibility of Data.** NF values are best comparable when obtained in a single stamping process, but nonetheless the absolute NF values are reproducible over independent exeriments. Here, mean NF values averaged over several measurements are displayed with their corresponding standard deviation. In measurements against an unmodified 40 bp duplex the nanobody-GFP interaction is much stronger in comparison resulting in very high NF values around 0.9.
(DOCX)

S2 Table **Original NF Data for the** **Figs. 2** **and** **3** **.** The orignal Normalized Fluorescence (NF) data with the corresponding standard deviation (SD) are given. For the data of Fig. 2, the difference between the respective NF values for Modified Enhancer and Enhancer is displayed, which increases the closer the NF values are to 0.5. The maximal deviation is calculated as the addition of the absolute values of the corresponding standard deviations.
(DOCX)
