# Correction: Parallel Force Assay for Protein-Protein Interactions

**DOI:** 10.1371/journal.pone.0119695

**Published:** 2015-03-20

**Authors:** The PLOS ONE Staff

There is an error in Equation 1. There should be a minus sign between the words RED and Ratio. Please view the correct equation below.

NF=(Ratio_RED–Ratio_FRET)/CE.
