# Supplementary material for: Parallel Force Assay for Protein-Protein Interactions
Source: PLoS One. 2014 Dec 29;9(12):e115049. doi: 10.1371/journal.pone.0115049 (PMC4278885; doi:10.1371/journal.pone.0115049)
Supplement: S1 Fig — Coupling of CoA-DNA to ybbR-tagged GFP. SDS-PAGE gel displaying the coupling between CoenzymeA-modified DNA to the ybbR-sfGFP construct in both fluorescence scans and Coomassie staining. In this sample gel, both GFP and CoA-DNA were mixed in equal concentrations (5 µM) as in the standard protocol [42]. (PDF) [file pone.0115049.s001.pdf]

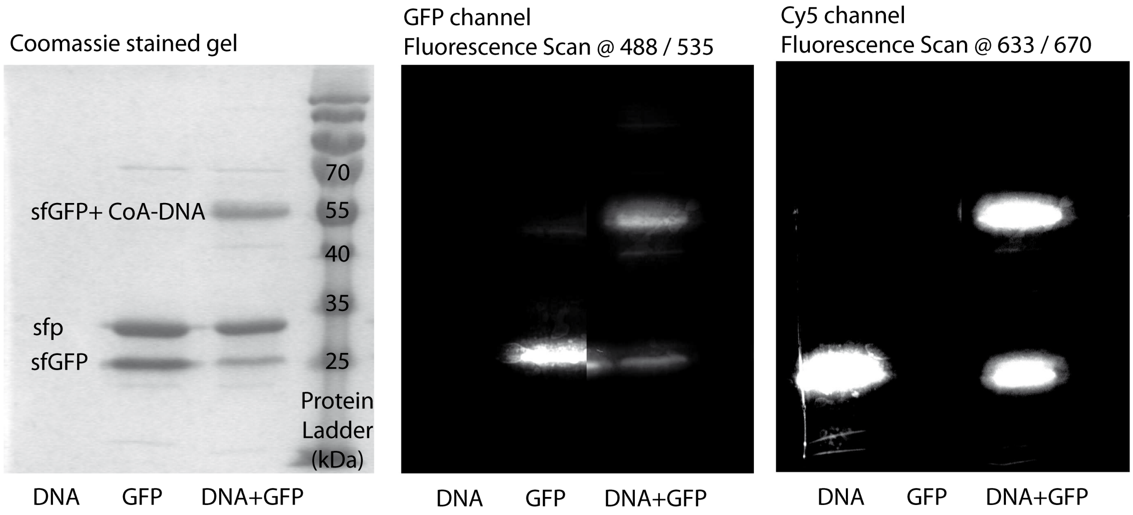

Figure S1. Coupling of CoA-DNA to ybbR-tagged GFP.

SDS-PAGE gel displaying the coupling between CoenzymeA-modified DNA to the ybbR-sfGFP construct in both fluorescence scans and Coomassie staining. In this sample gel, both GFP and CoA-DNA were mixed in equal concentrations (5µM) as in the standard protocol [1].

References

1. Yin J, Lin AJ, Golan DE, Walsh CT (2006) Site-specific protein labeling by Sfp phosphopantetheinyl transferase. Nat Protoc. pp. 280-285.
