# Supplementary material for: Parallel Force Assay for Protein-Protein Interactions
Source: PLoS One. 2014 Dec 29;9(12):e115049. doi: 10.1371/journal.pone.0115049 (PMC4278885; doi:10.1371/journal.pone.0115049)
Supplement: S2 Fig — DNA References. The reference DNA duplexes are displayed. The strand containing the CoenzymeA and Cy5 modification stays the same for all three types of reference, whereas the complementary strand modified with Cy3 and Biotin varies in length and constitution of bases. Chemical structures of the propynyl bases replacing their corresponding cytidine and thymidine bases are shown (structures provided by biomers.net GmbH, Germany). The polyamide ligands P1, (R)-P2 and (R)-P3 from [23] bind to the highlighted six base pair long binding sequence in the DNA reference duplex. (PDF) [file pone.0115049.s002.pdf]

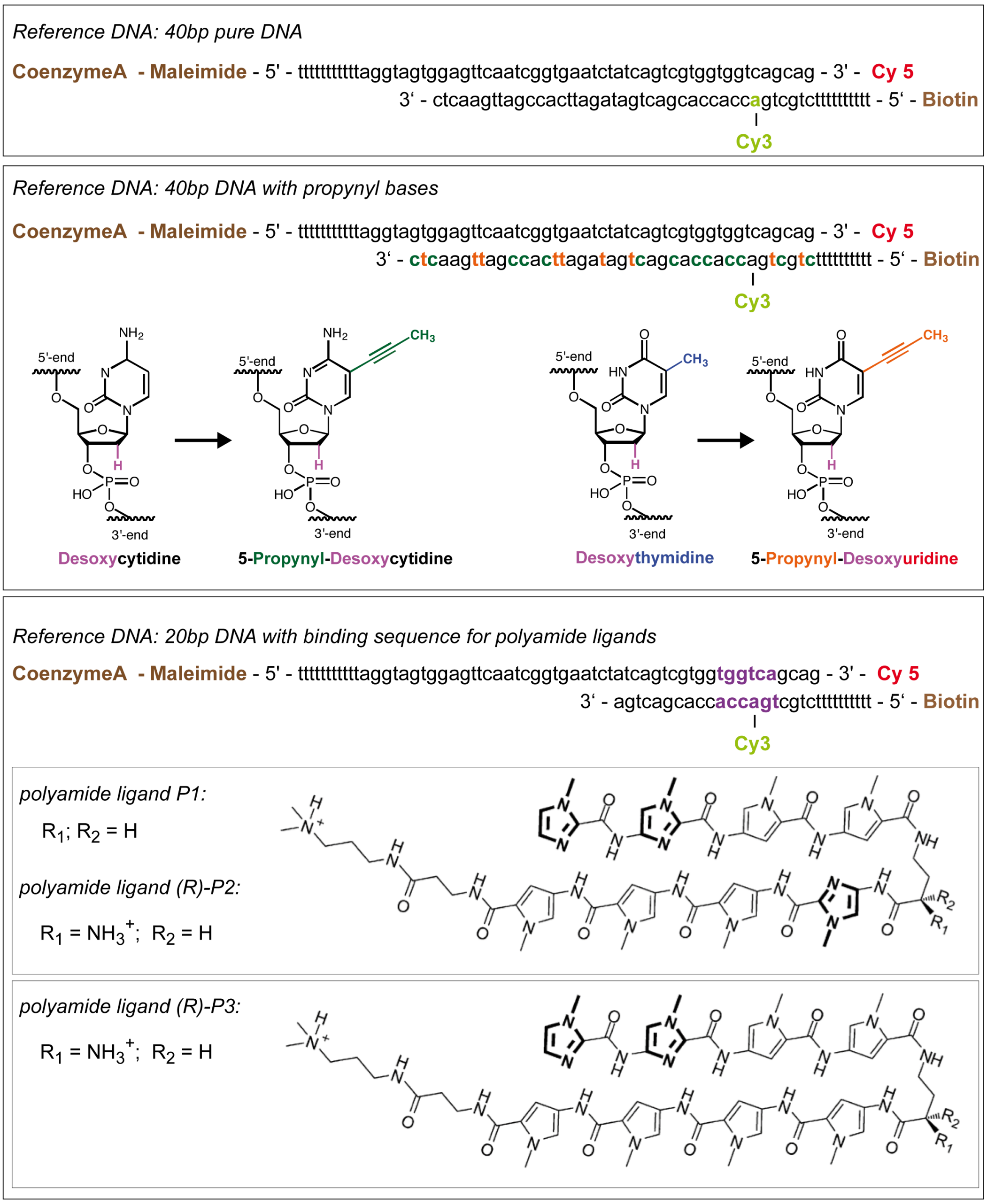

Figure S2. DNA References.

The reference DNA duplexes are displayed. The strand containing the CoenzymeA and Cy5 modification stays the same for all three types of reference, whereas the complementary strand modified with Cy3 and Biotin varies in length and constitution of bases. Chemical structures of the propynyl bases replacing their corresponding cytidine and thymidine bases are shown (structures provided by biomers.net GmbH, Germany). The polyamide ligands *P1*, *(R)-P2* and *(R)-P3* from [1] bind to the highlighted six base pair long binding sequence in the DNA reference duplex.

References

1. Ho D, Dose C, Albrecht CH, Severin P, Falter K, et al. (2009) Quantitative detection of small molecule/DNA complexes employing a force-based and label-free DNA-microarray. Biophys J. pp. 4661-4671.
