# Supplementary material for: Parallel Force Assay for Protein-Protein Interactions
Source: PLoS One. 2014 Dec 29;9(12):e115049. doi: 10.1371/journal.pone.0115049 (PMC4278885; doi:10.1371/journal.pone.0115049)
Supplement: S3 Fig — Sequence Alignment of the GFP Variants. The sequence alignment of all three variants of GFP displays the differences in the amino acid sequences and highlights the positions of the direct contacts (pink) and hydrophobic interactions (pale pink) to the nanobody Enhancer obtained for wtGFP by [30]. For eGFP, none of the interacting amino acids are mutated, but for sfGFP two of the contacts sites for Enhancer are different. In addition, all three amino acids forming the hydrophobic interaction are mutated. Sequence Alignment of GFPs was performed using Clustal W2 (http://www.ebi.ac.uk/Tools/msa/clustalw2/). (PDF) [file pone.0115049.s003.pdf]

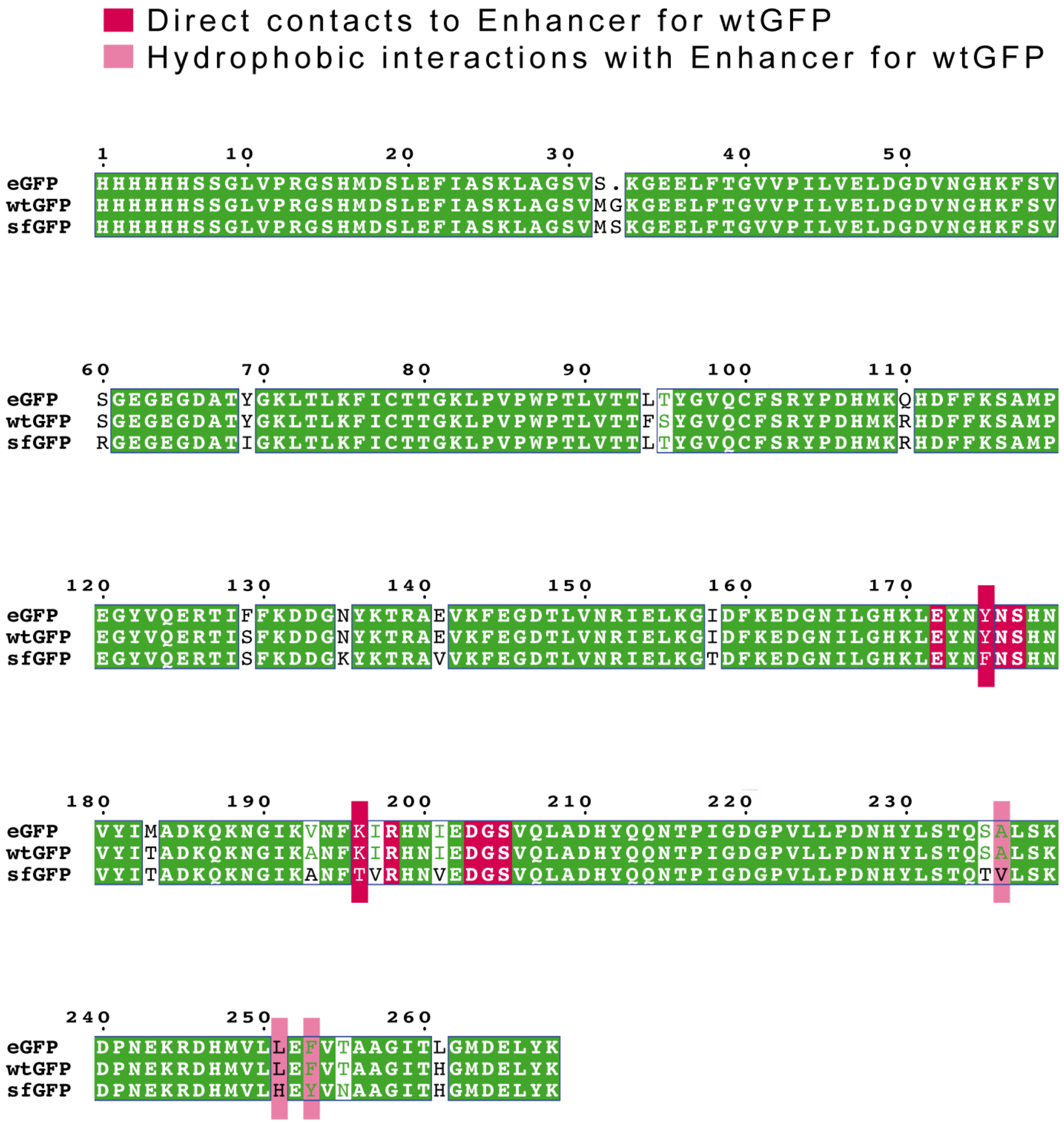

Figure S3. Sequence Alignment of the GFP Variants.

The sequence alignment of all three variants of GFP displays the differences in the amino acid sequences and highlights the positions of the direct contacts (pink) and hydrophobic interactions (pale pink) to the nanobody Enhancer obtained for wtGFP by [1]. For eGFP, none of the interacting amino acids are mutated, but for sfGFP two of the contacts sites for Enhancer are different. In addition, all three amino acids forming the hydrophobic interaction are mutated. Sequence Alignment of GFPs was performed using Clustal W2 (http://www.ebi.ac.uk/Tools/msa/clustalw2/).

References

1. Kirchhofer A, Helma J, Schmidthals K, Frauer C, Cui S, et al. (2010) Modulation of protein properties in living cells using nanobodies. Nat Struct Mol Biol. pp. 133-138.
