# Supplementary material for: Parallel Force Assay for Protein-Protein Interactions
Source: PLoS One. 2014 Dec 29;9(12):e115049. doi: 10.1371/journal.pone.0115049 (PMC4278885; doi:10.1371/journal.pone.0115049)
Supplement: S1 Supplement — Materials and Methods. (PDF) [file pone.0115049.s004.pdf]

Supplement S1. Materials and Methods.

Preparation of Proteins

Preparation of Nanobodies Enhancer and Modified Enhancer

Both nanobody constructs were cloned into a pHEN6 vector and harbor a pelB leader sequence for periplasmic export and a C-terminal Hexa-His-Tag for purification, followed by a terminal Cysteine for covalent, site directed coupling of the protein. For expression, a 5l E. coli JM109 culture was induced with 0.5mM isopropyl β-D-1-thiogalactopyranoside and incubated for 5 hours at 30°C. Cells were lysed by sonification in buffer containing 1xPBS pH 8.0, 0.5M NaCl, 20mM imidazole, 1mM PMSF and 10 g/l lysozyme. After centrifugation, the nanobody constructs in the soluble fraction were purified by immobilized metal affinity chromatography (IMAC) on prepacked 1ml HisTrap HP columns with an Äkta Explorer HPLC system (GE Healthcare, Freiburg, Germany) according to manufacturer's instructions. The elution fractions were analyzed by SDS-PAGE. Purified nanobody fractions were pooled and dialysed overnight into 1xPBS, flash-frozen and stored at -80°C at concentrations of 21µM (Modified Enhancer) and 35µM (Enhancer).

Nanobody Sequences

- Construct of Enhancer (PDB 3K1K) as in [1] with an additional C-terminal Cysteine:

QVQLVESGGALVQPGGSLRLSCAASGFPVNRYSMRWYRQAPGKEREWVAGMSSAGDRSSYEDSV
KGRFTISRDDARNTVYLQMNSLKPEDTAVYYCNVNVGFEYWGQGTQVTVSSHHHHHHC

- The construct of Modified Enhancer harbors an additional N-terminal, very positively charged, 12 amino acid long tag and a C-terminal Cysteine:

GRKKRRQRRRGSQVQLVESGGALVQPGGSLRLSCAASGFPVNRYSMRWYRQAPGKEREWVAGM
SSAGDRSSYEDSVKGRFTISRDDARNTVYLQMNSLKPEDTAVYYCNVNVGFEYWGQGTQVTVSSHH
HHHHC

Preparation of GFPs

All GFP constructs were designed to harbor an N-terminal Hexa-His-Tag for purification, followed by the ybbR-tag (DSLEFIASKLA) [2,3] and the respective GFP type (wtGFP, eGFP and sfGFP; for the sequences see the alignment in Figure S3). All fusion proteins were cloned into pET28a vectors (EMD Group, Merck KGaA, Darmstadt, Germany) and were expressed in *E.coli* BL21 DE3 CodonPlus cells (Agilent Technologies, Inc., Santa Clara, CA, USA). For this, 0.5l of SB medium was inoculated with 5ml of an over night culture and grown at 37°C until an OD_{600} of around 0.7 had been reached. Then, over night expression at 18°C was induced by the addition of 0.25mM IPTG. Cells were lysed by sonification in 50mM Tris pH 7.5, 100mM NaCl, 5% Glycerin, 15mM Imidazole and 10mM β-Mercaptoethanol. After centrifugation the ybbR-GFP constructs in the soluble fraction were purified by immobilized metal affinity chromatography (IMAC) on prepacked 1ml HisTrap HP columns with an Äkta Explorer HPLC system (GE Healthcare, Freiburg, Germany) according to manufacturer's instructions. The elution fractions were analyzed by SDS-PAGE and pooled accordingly. The pooled protein samples were then dialyzed into storage buffer (30mM Tris pH 7,5, 100mM NaCl, 5% Glycerin, 2mM DTT) over night, and stored at -80°C at final concentrations of 50µM for sfGFP, 550µM for eGFP and 200µM for wtGFP.

Protein-DNA coupling

In general, the Phosphopantetheinyl Transferase (Sfp)-mediated coupling of CoenzymeA modified DNA to ybbR-tagged proteins offers a very high yield. A standard protocol for the coupling reaction can be found in [2]. The fraction of reacted GFP or DNA can be tuned by adjusting the respective concentrations. In the experiments conducted here, a high fraction of reacted GFP was desired, so that most GFPs binding to the nanobodies carry a DNA reference and thus form a fully functional Molecular Force Probe. In a slightly altered coupling reaction, first the DNA duplex was hybridized by mixing the CoA strand in a ratio of 1:2 with the biotinylated strand (to again ensure that the CoA strands form a duplex). This pre-incubated mix containing 10µM CoA-DNA was then combined with 5µM of the corresponding GFP sample and 6,65µM Sfp in a final 10µl Ansatz in Sfp buffer (150mM NaCl, 1mM DTT, 10mM MgCl and 50mM Tris) and used after incubation at room temperature for at least 1 hour.

The DNA oligonucleotides were purchased, including all modifications, from biomers.net GmbH, Germany.

Chemical Procedures

Assembly of Protein-MFPs

Microscopy glass slides were aminosilanized in our lab (for a detailed protocol, see eg. [4]) and deprotonated in sodium borate buffer (50mM H_3BO_3, 50mM $Na_2B_4O_7$•10 H_2O pH=8.5) for 45 minutes. For functionalization, 50mM NHS-PEG-Maleimide crosslinker (MW 5000; Rapp-Polymere, Germany) in sodium borate buffer was incubated for 1 hour. After careful drying of the slide with N_2 gas, a custom-made silicone isolator with 16-wells in a 4x4 array (Grace-Biolabs, USA) was placed on the glass slide. To obtain free Cysteines at the C- termini of the nanobodies, possible intermolecular disulfide bonds were reduced with TCEP
beads (Immobilized TCEP Disulfide Reducing Gel, Thermo Fisher Scientific inc., Rockford, IL, USA) for 30 min. After removal of the beads, samples were spun down in a table top centrifuge for 15 min to remove agglomerates. The supernatant with the respective nanobody was pipetted in the wells of the isolator and incubated for 1 hour. The wells were then rinsed thoroughly with 1xPBS and the respective GFP-DNA constructs (for preparation see: Protein-DNA coupling) were spotted into the wells for incubation of 1 hour. To remove unbound free DNA and Protein-DNA constructs, the slide was rinsed in washing steps with 2x, 0.2x and finally 1xPBS, which acts as buffer for the measurement. Care was taken to ensure aqueous buffer environment for the samples at all time during the preparation process. In measurements with polyamide, 1µM of ligand was added to the measurement buffer and left to incubate for 2 hours before measurement. In general, all samples were measured within 3 hours after sample preparation.
Note, that the temporal and spatial delimitation of the probe assembly would also allow for surface immobilization *via* a ybbR-tag, if thiol-chemistry were unfavorable. In this case, a purification of the DNA-protein complexes is necessary to remove the Sfp.

Stamp preparation

Fabrication and functionalization of the PDMS (polydimethylsiloxane) stamp has been described in detail elsewhere (*e.g.* in [5,6]). In brief, 1:10 of crosslinker/base (Sylgard, Dow Corning, MI, USA) was cast in a custom-made micro- and macrostructured Pyrex/silicon wafer (HSG-IMIT, Germany) according to standard procedures. They were then cut into an arrangement of 4x4 pillars, so that the final stamps feature 16 pillars of 1mm in height and 1.1mm in diameter on a 3mm thick basis. The top

of the pillars is microstuctured with pads of 100µm x 100xµm separated by trenches (41 µm in width, 5 µm in depth) to ensure liquid drainage during the contact and separation process.

For the functionalization, the stamps were activated in 12.5% hydrochloric acid over night and derivatized with (3- glycidoxypropyl)-trimethoxysilane (ABCR, Karlsruhe, Germany) for the generation of epoxide groups. A 1:1 mix of NH_2-PEG-Biotin (MW 3400) and NH_2-PEG-CH_3 (MW 2000) (Rapp-Polymere, Germany) was melted at 80°C, about 1µl was spotted to each pillar and incubated over night at 80°C under argon. The excess polymers were thoroughly removed by rinsing with ddH_20. For final functionalization, the stamps were incubated for 60 min with 1xPBS containing 0.4% (w/v) BSA and 1 mg/ml Streptavidin (Thermo Fisher Scientific, Bonn, Germany), rinsed with 0.05% Tween 20 (VWR Scientific GmbH, Germany) in 0.2xPBS and gently dried with N_2 gas.

Measurement and Analysis

As the measurement process and the pixel-by-pixel analysis are identical to that of the original DNA-MFA, additional information to the explanations in the main text can be found in the corresponding publication of Severin *et.al.* [7].

References

1. Kirchhofer A, Helma J, Schmidthals K, Frauer C, Cui S, et al. (2010) Modulation of protein properties in living cells using nanobodies. Nat Struct Mol Biol. pp. 133-138.
2. Yin J, Lin AJ, Golan DE, Walsh CT (2006) Site-specific protein labeling by Sfp phosphopantetheinyl transferase. Nat Protoc. pp. 280-285.
3. Yin J, Straight PD, McLoughlin SM, Zhou Z, Lin AJ, et al. (2005) Genetically encoded short peptide tag for versatile protein labeling by Sfp phosphopantetheinyl transferase. Proc Natl Acad Sci USA. pp. 15815-15820.
4. Limmer K, Pippig DA, Aschenbrenner D, Gaub HE (2014) A Force-Based, Parallel Assay for the Quantification of Protein-DNA Interactions. PLoS ONE. pp. e89626.
5. Limmer K, Aschenbrenner D, Gaub H (2013) Sequence-specific inhibition of Dicer measured with a force-based microarray for RNA ligands. Nucleic Acids Res.
6. Severin PMD, Gaub HE (2012) DNA-Protein Binding Force Chip. Small.
7. Severin PMD, Ho D, Gaub HE (2011) A high throughput molecular force assay for protein-DNA interactions. Lab Chip. pp. 856-862.
