# Supplementary material for: Parallel Force Assay for Protein-Protein Interactions
Source: PLoS One. 2014 Dec 29;9(12):e115049. doi: 10.1371/journal.pone.0115049 (PMC4278885; doi:10.1371/journal.pone.0115049)
Supplement: S1 Table — Reproducibility of Data. NF values are best comparable when obtained in a single stamping process, but nonetheless the absolute NF values are reproducible over independent exeriments. Here, mean NF values averaged over several measurements are displayed with their corresponding standard deviation. In measurements against an unmodified 40 bp duplex the nanobody-GFP interaction is much stronger in comparison resulting in very high NF values around 0.9. (DOCX) [file pone.0115049.s005.docx]

**Table S1. Reproducibility of Data.**

NF values are best comparable when obtained in a single stamping process, but nonetheless the absolute NF values are reproducible over independent exeriments. Here, mean NF values averaged over several measurements are displayed with their corresponding standard deviation.

In measurements against an unmodified 40bp duplex the nanobody-GFP interaction is much stronger in comparison resulting in very high NF values around 0.9.
