# Supplementary material for: Parallel Force Assay for Protein-Protein Interactions
Source: PLoS One. 2014 Dec 29;9(12):e115049. doi: 10.1371/journal.pone.0115049 (PMC4278885; doi:10.1371/journal.pone.0115049)
Supplement: S2 Table — Original NF Data for the Figs. 2 and 3 . The orignal Normalized Fluorescence (NF) data with the corresponding standard deviation (SD) are given. For the data of Fig. 2, the difference between the respective NF values for Modified Enhancer and Enhancer is displayed, which increases the closer the NF values are to 0.5. The maximal deviation is calculated as the addition of the absolute values of the corresponding standard deviations. (DOCX) [file pone.0115049.s006.docx]

**Table S2. Original NF Data for the Figures 2 and 3.**

The orignal Normalized Fluorescence (NF) data with the corresponding standard deviation (SD) are given. For the data of Figure 2, the difference between the respective NF values for Modified Enhancer and Enhancer is displayed, which increases the closer the NF values are to 0.5. The maximal deviation is calculated as the addition of the absolute values of the corresponding standard deviations.
